# The study of electronic nematicity in an overdoped (Bi, Pb)_2_Sr_2_CuO_6+δ_ superconductor using scanning tunneling spectroscopy

**DOI:** 10.1038/s41598-017-08376-1

**Published:** 2017-08-14

**Authors:** Yuan Zheng, Ying Fei, Kunliang Bu, Wenhao Zhang, Ying Ding, Xingjiang Zhou, Jennifer E. Hoffman, Yi Yin

**Affiliations:** 1 Department of Physics, Zhejiang University, Hangzhou, 310027 China; 2 Beijing National Laboratory for Condensed Matter Physics, Institute of Physics, Academy of Science, Beijing, 100190 China; 3 Collaborative Innovation Center of Quantum Matter, Beijing, 100871 China; 4 Department of Physics, Harvard University, 17 Oxford St., Cambridge, 02138 USA; 5 Collaborative Innovation Center of Advanced Microstructures, Nanjing, 210093 China

## Abstract

The pseudogap (PG) state and its related intra-unit-cell symmetry breaking remain the focus in the research of cuprate superconductors. Although the nematicity has been studied in Bi_2_Sr_2_CaCu_2_O_8+δ_, especially underdoped samples, its behavior in other cuprates and different doping regions is still unclear. Here we apply a scanning tunneling microscope to explore an overdoped (Bi, Pb)_2_Sr_2_CuO_6+δ_ with a large Fermi surface (FS). The establishment of a nematic order and its real-space distribution is visualized as the energy scale approaches the PG.

## Introduction

The electronic nematic order has attracted a lot of interests in both the cuprate and iron-based high-transition-temperature (high-*T*_*c*_) superconductors^1–3^. In solid state systems with the nematic order, the rotational symmetry is broken while the translational lattice symmetry is preserved^4^. Various experimental techniques, such as x-ray scattering, neutron scattering, angle-resolved photoemission spectroscopy (ARPES), and scanning tunneling microscope (STM), have been applied to determine the nematicity through the evidences of symmetry breaking in orbital and magnetic orders^5–8^. The understanding about nematic order may help unravel the mechanism of the superconducting (SC) state^9–11^.

STM is a powerful tool for detecting electronic structures with an atomic resolution in the real space. For Bi_2_Sr_2_CaCu_2_O_8+δ_ (Bi-2212), the STM studies have revealed a 90º rotational symmetry breaking for the O sites within each CuO_2_ unit cell, around the energy level of the pseudogap (PG) states^4^. The nematicity is suggested to be a relevant order parameter of the PG state which strongly intertwines with the SC state. The STM studies of Bi-2212 have further clarified that the nematic order in general decreases with doping and finally disappears when a small-to-large Fermi surface (FS) reconstruction occurs at carrier doping of *p*~0.19^12^.

With similar phase diagrams, various cuprate superconductors can differ in the detailed electronic structures^13, 14^. As the doping increases, the small-to-large FS transition also occurs in (Bi, Pb)_2_Sr_2_CuO_6+δ_ (Bi-2201) at *p*~0.15. However, the PG phase extends to the overdoped regime for Bi-2201^14, 15^ while terminating in the SC dome for Bi-2212. It is thus physically interesting to investigate the nematic order in overdoped Bi-2201 where the PG state coexists with a large FS. The study in this regime may enable us to explore the early stage in the formation of the nematicity. In this paper, we apply STM to study an overdoped Bi-2201 sample through an analysis approach similar to that in ref. 4. Our studies confirm the establishment of the nematic order and reveal a strong real-space fluctuation of site-specified order parameters.

## Results

### Topographical and electronic properties of overdoped Bi-2201

**Figure 1:**
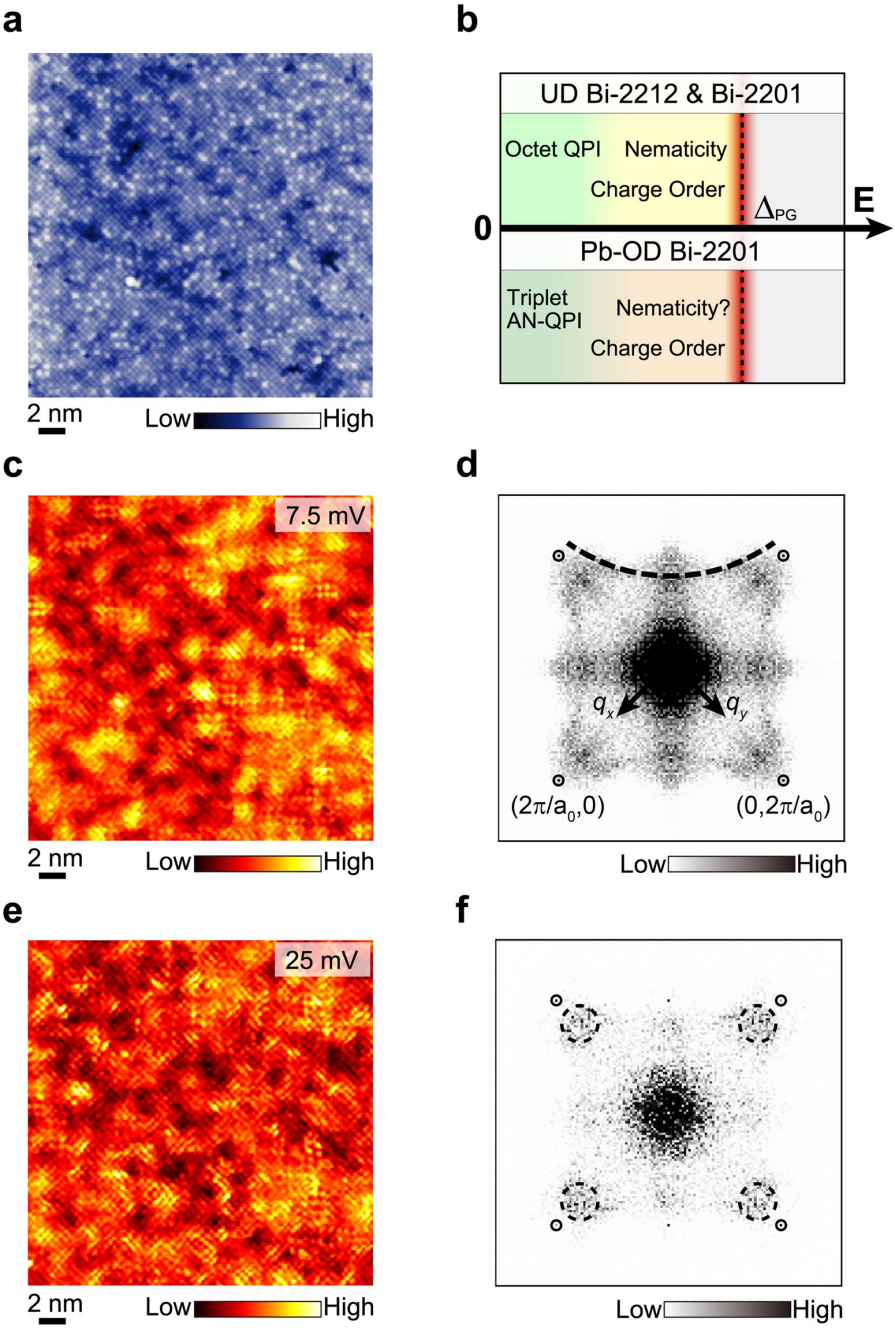
Topography and electronic properties of an overdoped Bi-2201 (*T*_*c*_ = 13 K). (**a**) Topographical image (270 × 270 Å^2^) of a cleaved BiO layer with the bias voltage *V* = 100 mV and tunneling current *I* = 100 pA. The brighter spots in the lattice correspond to the Pb substitutes. (**b**) A diagram of different electronic behaviors, evolving as the energy increases from the Fermi energy (zero) to the PG energy Δ_PG_. (**c**) A differential tunneling conductance *dI*/*dV* map measured at *V* = 7.5 mV on the same FOV as in (**a**). (**d**) The *dI*/*dV* map in the momentum space after the Fourier transform of panel c. The four Bragg peaks are labelled in the small circles. The dashed arc represents a large FS. (**e**) Another *dI*/*dV* map measured at *V* = 25 mV on the same FOV as in (**a**). (**f**) The *dI*/*dV* map in the momentum space after the Fourier transform of panel e. The four peaks representing a charge order are enclosed in the dashed circles inside the first Brillouin zone near Bragg peaks.

The samples studied in this paper are overdoped (Bi, Pb)_2_Sr_2_CuO_6+δ_ single crystals with *T*_*c*_ = 13 K and all the data are taken in an ultra-high-vacuum STM at *T* = 4.5 K. Figure 1a displays a topographic image obtained on a cleaved BiO surface, showing a clear square lattice of Bi atoms with interatomic spacing of *a*_0_ ≈ 3.8 Å. A part of Bi atoms are substituted by Pb atoms, represented by brighter spots in the lattice, and the incommensurate supermodulation is completely suppressed due to the elimination of the periodic potential of strain^16^.

The local electronic property can be probed by the differential conductance (*dI*/*dV*) spectroscopy, which is proportional to the local density of states (LDOS). A spectral survey is taken simultaneously with the topographic image in Fig. 1a, at a dense array of locations. All the following data are shown and analyzed in the same field of view (FOV) and the atomic registry is precisely maintained. Figure 1c presents a typical slice of the spectroscopy image or a differential conductance map, $g(\overrightarrow{r},\,E)$, at a relative low bias voltage *V* = 7.5 mV. The wavelike spatial pattern of the LDOS modulation mainly originates from the Bogoliubov quasi-particle interference (QPI)^17, 18^. In the Fourier transformed map, $\tilde{g}(\overrightarrow{q},E=7.5\,{\rm{meV}})$ (see Fig. 1d), the dominant QPI wave vectors extracted around strong signals could provide the Fermi surface (FS) information. We observe a ‘triplet’ signal in the anti-nodal region and a trace extending from the nodal to anti-nodal regions. These two features are assigned as the signature of a large FS^14^. As a comparison, an ‘octet’ QPI associated with a small FS has been observed for underdoped Bi-2212 and Bi-2201 samples (see Fig. 1b)^13, 14, 17^.

The Bogoliubov quasi-particle excitations, which is related with the SC phase, are often detected around the Fermi energy. The electronic excitations behave differently as the energy increases to the PG energy scale^13^. In Fig. 1e, we present another $g(\overrightarrow{r},\,E)$ at a relative high bias voltage *V* = 25 mV. The wavelike pattern here is not from the QPI, but related with a quasi-localized charge order (also called the smectic order)^4, 9^. In the Fourier transformed map, $\tilde{g}(\overrightarrow{q},\,\,E=25\,{\rm{meV}})$ (see Fig. 1f), the dominant wave vectors are near ${\overrightarrow{q}}^{\ast }\sim \pm \,\frac{3}{4}{\overrightarrow{Q}}_{x}$ and $\pm \,\frac{3}{4}{\overrightarrow{Q}}_{y}$, corresponding to the real-space periodicity of charge order modulation. Here $\pm {\overrightarrow{Q}}_{x}=(\pm \frac{2\pi }{{a}_{0}},0)$ and $\pm {\overrightarrow{Q}}_{y}=(0,\,\pm \frac{2\pi }{{a}_{0}})$ are positions of Bragg peaks. Although both the intra-unit-cell nematicity and the charge order are developed when the energy approaches the PG magnitude (see Fig. 1b), these two orders are prominent at different wave vectors in the Fourier transformed map. The former is around the Bragg peaks, while the latter is around ${\overrightarrow{q}}^{\ast }$ ^4, 9^.

### Collective nematic order

**Figure 2:**
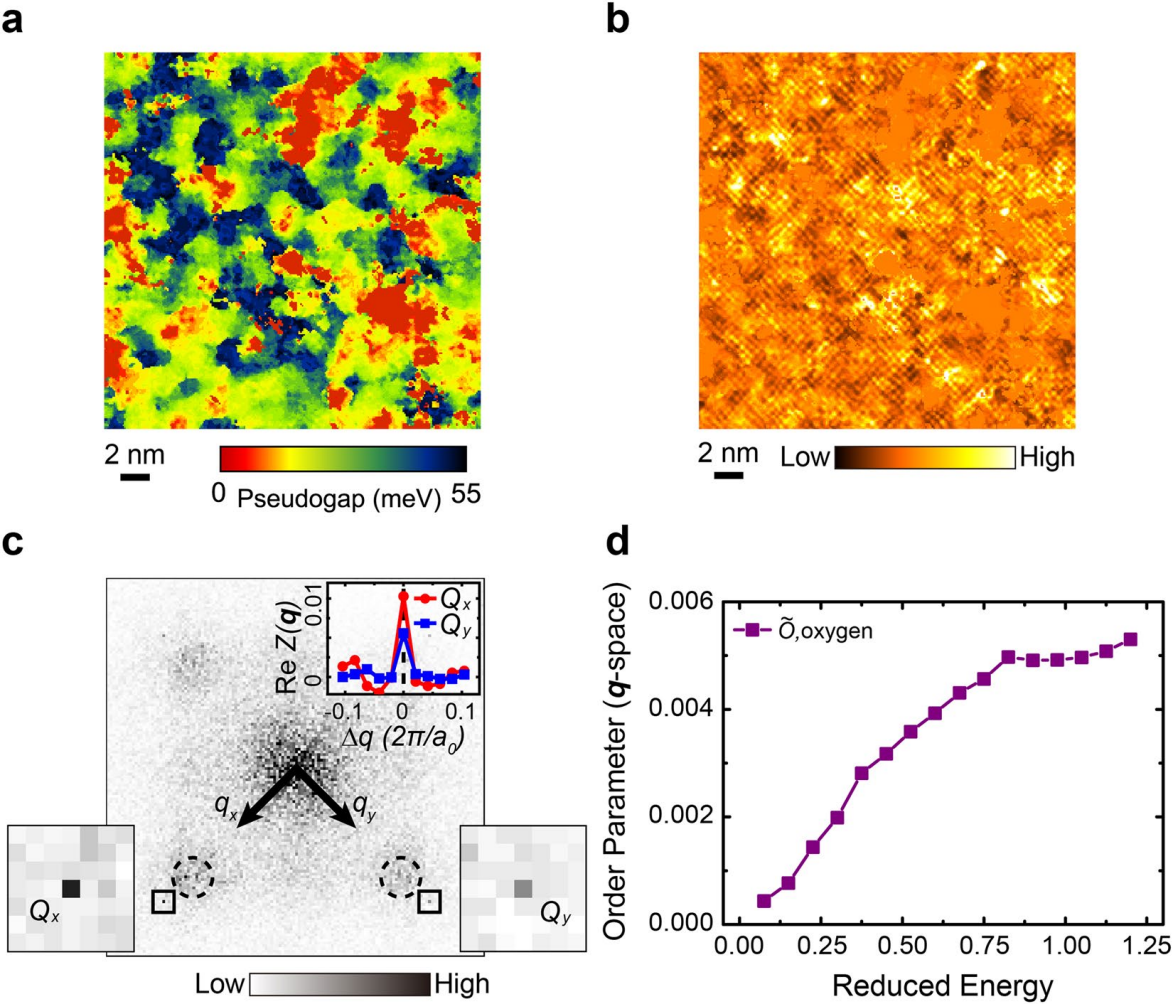
Ratio *Z*-map and collective nematic order (*q*-space). (**a**) The PG map in the same FOV as in Fig. 1a. (**b**) A typical $Z(\overrightarrow{r},\varepsilon )$ map at the reduced energy of *ε* = 0.975, with the VHS regions filled with the spatial average value. (**c**) The Fourier transform $\tilde{Z}(\overrightarrow{q},\varepsilon )$ map of panel b in the momentum space. For the four Bragg peaks, the positions at *Q*_*x*_ and *Q*_*y*_ are highlighted and enlarged, in which both Bragg peaks collapse into one pixel. In the inset, the real parts of the line cuts at *Q*_*x*_ and *Q*_*y*_ are shown in red and blue colors, respectively. The charge order is prominent at different wave vectors, as labeled by the dashed circles. (**d**) The collective order parameter as a function of *ε* in the momentum space.

Although the current Bi-2201 sample is overdoped with a large FS, the PG state is known to exist^14, 15^. The PG magnitude (Δ_PG_) at each location in the FOV is determined by extracting the bias voltage of the positive coherence peak from its *dI*/*dV* spectroscopy. The resulting PG map in Fig. 2a shows a strong nanoscale inhomogeneity where large-Δ_PG_ regions are spread in space and surrounded by low-Δ_PG_ regions. In the broad distribution of Δ_PG_, we also find ~10.9% zero gap patches, which are attributed to van Hove singularities (VHS, with details in Supplementary Note S1 and Fig. S1). The similar VHS behavior was specified in pure non-cation-doped Bi-2201 samples^19^. To detect the electronic order hidden by the strong inhomogeneity of the PG distribution, we apply a ratio map, $Z(\overrightarrow{r},\varepsilon )=g(\overrightarrow{r},\varepsilon )/g(\overrightarrow{r},-\varepsilon )$ ^20^. The reduced energy $\varepsilon =|E|/{\Delta }_{{\rm{PG}}}(\overrightarrow{r})$ is rescaled with respect to the PG magnitude at each location. The ratio $Z(\overrightarrow{r},\varepsilon )$ between the LDOS at two opposite reduced energies (±*ε*) can minimize the systematic error of $g(\overrightarrow{r},\pm \varepsilon )$ caused by the setpoint effect^20^.

Figure 2b displays a typical image of $Z(\overrightarrow{r},\varepsilon =0.975)$ in the same atomically resolved area as in Fig. 1a. The spatial average of $Z(\overrightarrow{r},\varepsilon )$ is artificially assigned for the VHS regions (see Supplementary Note S1 and Fig. S1), which however does not affect the main conclusion of this paper. The electronic spatial patterns consist of an apparent charge order modulation and an underlying intra-unit-cell order. To obtain an atomic registry with picometer-scale precision, we implement a lattice drift correction of the topographic image in all the *dI*/*dV* maps simultaneously acquired^4, 21^. After the lattice drift correction, we implement the Fourier transform and obtain a ratio map, $\tilde{Z}(\overrightarrow{q},\varepsilon =0.975)$, in the momentum space (Fig. 2c). Four sharp Bragg peaks at $\pm {\overrightarrow{Q}}_{x}$ and $\pm {\overrightarrow{Q}}_{y}$ are observed, each collapsing into a single pixel due to the drift correction. However, the two sets of Bragg peaks are not degenerate as revealed by the difference between $\mathrm{Re}\,\tilde{Z}(\pm {\overrightarrow{Q}}_{x},\varepsilon =0.975)$ and $\mathrm{Re}\,\tilde{Z}(\pm {\overrightarrow{Q}}_{y},\varepsilon =0.975)$ (shown in the inset of Fig. 2c). As a Cu site is selected as the origin when performing the Fourier transform, this difference signifies a symmetry breaking from *C*_4*v*_ (90° rotational symmetry for four O sites surrounding each Cu site) to *C*_2*v*_ (180° rotational symmetry for two O sites along the *x*/*y* direction). A collective nematic order for this FOV of overdoped Bi-2201 is thus determined around the PG energy scale. The same investigation is applied to other reduced energies, which leads to the definition of a normalized order parameter,

1 $$\tilde{O}(\varepsilon )=[\mathrm{Re}\,\tilde{Z}({\overrightarrow{Q}}_{x},\varepsilon )-\mathrm{Re}\,\tilde{Z}({\overrightarrow{Q}}_{y},\varepsilon )]/\bar{Z}(\varepsilon ),$$

where $\bar{Z}(\varepsilon )$ is the spatial average of $Z(\overrightarrow{r},\varepsilon )$. In Fig. 2d, the order parameter $\tilde{O}({\rm{\varepsilon }})\,$ increases monotonically as *ε* approaches the PG energy, illustrating a gradual establishment of the nematic order.

**Figure 3:**
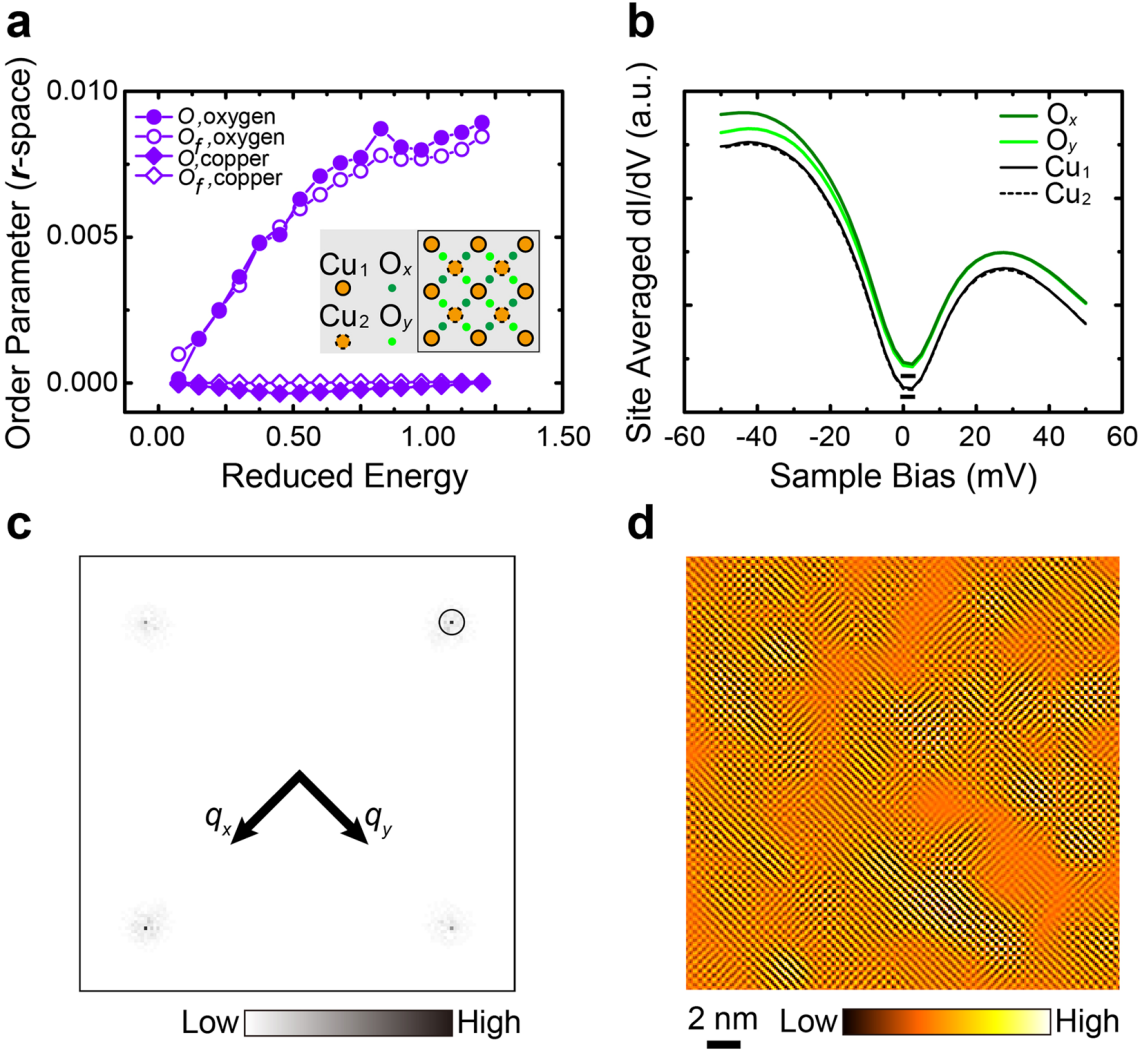
Filtered *Z*-map and collective nematic order (*r*-space). (**a**) The collective order parameter in the real space as a function of *ε*. The lines with filled circles and diamonds are the results of *O*(*ε*) from the O and Cu sites, respectively. The lines with open circles and diamonds are the results of *O*_*f*_(*ε*) from the same two types of sites after the filtration. The inset is a schematic of CuO_2_ layer. (**b**) The averaged spectra on four different types of atomic sites (O_*x*_, O_*y*_, Cu_1_, and Cu_2_ as labeled in the inset of panel a). The spectra for O sites are offset vertically for clarity. (**c**) The filtered ratio ${\tilde{Z}}_{f}(\overrightarrow{q},\varepsilon )$ map in the momentum space after a Gaussian filtration around the four Bragg peaks. A circle centered at *Q*_*x*_ with the radius *Λ* ^−1^ = 1.05 nm is drawn to show the filtering size. (**d**) The filtered ratio ${Z}_{f}(\overrightarrow{r},\varepsilon )$ map after the inverse Fourier transform of panel c.

The information extracted from the Bragg peaks in the momentum space represents an asymmetric intra-unit-cell electronic modulation along the *x* and *y* directions in the real space. Based on the spatial structure shown in the inset of Fig. 3a, the dominant order in $\mathrm{Re}\,\tilde{Z}(\pm {\overrightarrow{Q}}_{x},\varepsilon )$ and $\mathrm{Re}\,\tilde{Z}(\pm {\overrightarrow{Q}}_{y},\varepsilon )$ arises from the O sites. In the real space ratio map of $Z(\overrightarrow{r},\varepsilon )$, we take a summation over the unit cells and calculate an alternative order parameter^4^,

2 $$O(\varepsilon )={\sum }_{n}O(n,\,\varepsilon )/N={\sum }_{n}[{Z}_{y}({\overrightarrow{r}}_{n},\varepsilon )-{Z}_{x}({\overrightarrow{r}}_{n},\varepsilon )]/[N\bar{Z}(\varepsilon )],$$

where ${\overrightarrow{r}}_{n}$ is the location of the Cu site centered in the *n*th unit cell, and *N* is the total number of unit cells. The results of ${Z}_{x}({\overrightarrow{r}}_{n},\varepsilon )$ and ${Z}_{y}({\overrightarrow{r}}_{n},\varepsilon )$ are averaged from the O sites of the *n*th unit cell along the *x* and *y* directions (labeled by O_*x*_ and O_*y*_), respectively. As the reduced energy *ε* increases, *O*(*ε*) follows the same trend as $\tilde{O}(\varepsilon )$, and a smooth transition from *C*_4*v*_ to *C*_2*v*_ is observed (as shown in Fig. 3a). The nematicity is a manifest from the asymmetric electronic structures on the O_*x*_ and O_*y*_ sites. As a comparison, we assign two set of copper sites, Cu_1_ and Cu_2_, along the two perpendicular directions, and the symmetry breaking of Cu sites is not found over the whole range of *ε*.

The nematicity defined in (2) is explored in the $Z(\overrightarrow{r},\varepsilon )$ maps which are derived from the *dI*/*dV* spectra. To confirm that the nematicity is not artificially induced by our data analysis, we directly average the *dI*/*dV* spectrum over all the O_*x*_/O_*y*_ sites in the FOV. As shown in Fig. 3b, the O_*x*_/O_*y*_-averaged spectra are distinguished from each other in the regime of negative bias voltages. The dataset analyzed above was taken with a tunnel junction of sample bias *V*_*b*_ = +100 mV. Since the spectral weight is normalized for empty states (positive bias voltages), the spectral shift from asymmetric nematic order appears in the filled states (negative bias voltages). Under the condition of *V*_*b*_ = −100 mV, we find the spectral shift of the O_*x*_/O_*y*_-averaged *dI*/*dV* spectra in the empty states (see Supplementary Information Note S3 and Fig. S4). The symmetry breaking of O_*x*_ and O_*y*_ sites is a result of the asymmetric LDOS while the ratio *Z*-map provides a clearer identification of the nematic order parameter. Instead, the averaged *dI*/*dV* spectra from Cu_1_ and Cu_2_ sites are indistinguishable over the whole energy range, which is consistent with the zero nematicity of Cu sites in Fig. 3a.

### Real-space distribution of the nematic order

The two order parameters, $\tilde{O}({\rm{\varepsilon }})$ and *O*(ε), reflect the same phenomenon of the nematic order, despite the fact that they are defined separately in the momentum and real spaces. The real-space $Z(\overrightarrow{r},\varepsilon )$ map will be further applied to explore the spatial distribution of nematicity. However, the electronic modulations other than the intra-unit-cell periodicity lead to strong interference signal in the real space, and the atomic-scale nematic order is buried in background noise and other electronic orders. A solution to this difficulty is to retain $\tilde{Z}(\overrightarrow{q},\varepsilon )$ within a limited region around four Bragg peaks in the momentum space, which can be realized by

3 $${\tilde{Z}}_{f}(\overrightarrow{q},\varepsilon )=\tilde{Z}(\overrightarrow{q},\varepsilon )[{f}_{\Lambda }(\overrightarrow{q}+{\overrightarrow{Q}}_{x})+{f}_{\Lambda }(\overrightarrow{q}-{\overrightarrow{Q}}_{x})+{f}_{\Lambda }(\overrightarrow{q}+{\overrightarrow{Q}}_{y})+{f}_{\Lambda }(\overrightarrow{q}-{\overrightarrow{Q}}_{y})].$$

The Gaussian filtering function, ${f}_{\Lambda }(\overrightarrow{q})=\exp (-{q}^{2}/2{\Lambda }^{2})$, is defined with a filtering size *Λ* ^−1^. Figure 3c presents a typical result of ${\tilde{Z}}_{f}(\overrightarrow{q},\varepsilon =0.975)$ filtered from Fig. 2c using *Λ* ^−1^ = 1.05 nm. The inverse Fourier transform is subsequently applied to ${\tilde{Z}}_{f}(\overrightarrow{q},\varepsilon =0.975)$ for a real-space map of ${Z}_{f}(\overrightarrow{r},\varepsilon =0.975)$, as shown in Fig. 3d. Compared to the original map in Fig. 2b, this filtered map reduces longer-wavelength modulations while the spatial inhomogeneity is partially preserved. In the real space, the filtration is equivalent to a locally weighted average for each spatial location so that the background signal can be significantly suppressed. Following the definition in Eq. (2), we calculate the order parameter *O*_*f*_(*ε*) after the filtration, and the comparison with *O*(*ε*) in Fig. 2a reveals the same development of the nematicity. Although the measurements of the collective order, *O*_*f*_(*ε*) and *O*(*ε*), are similar, the filtering procedure is important for detecting the spatial distribution of nematic order. For each *n*th unit cell, the difference between the electronic structures of the O sites leads to the order parameter of this cell,

4 $${O}_{f}(n,\varepsilon )=[{Z}_{f,y}({\overrightarrow{r}}_{n},\varepsilon )-{Z}_{f,x}({\overrightarrow{r}}_{n},\varepsilon )]/{\bar{Z}}_{f}(\varepsilon ),$$

where ${Z}_{f,x}({\overrightarrow{r}}_{n},\varepsilon )$ and ${Z}_{f,y}({\overrightarrow{r}}_{n},\varepsilon )$ are averaged over the O sites along the *x* and *y* directions. A coarse-grained average within the area of *Λ* ^−2^ is implied in our cell order parameter, which is consistent with a previous approach of estimating the correlation length by a local Fourier transform^4^. Consequently, we construct a new map of the cell nematic order parameter *O*_*f*_ *(n*,*ε*). The image resolution of *O*_*f*_ *(n*,*ε*) is reduced compared to that of $Z(\overrightarrow{r},\varepsilon )$, as the information of each unit cell is compressed into a single pixel.

**Figure 4:**
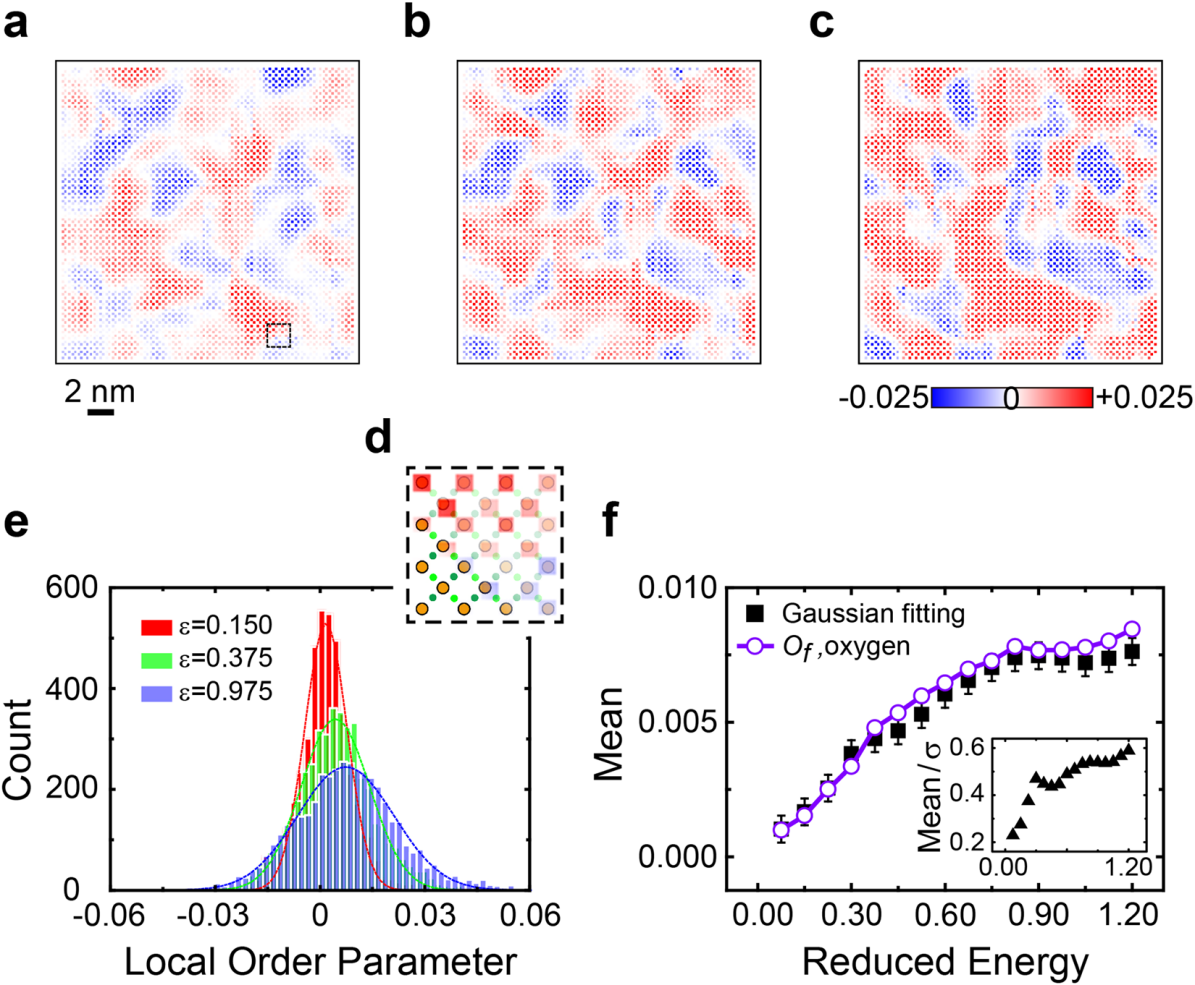
Distribution of Local nematic orders. Maps of cell order parameters *O*_*f*_(*n*,*ε*) in the real space with the reduced energies of (**a**) ε = 0.150, (**b**) ε = 0.375, and (**c**) ε = 0.975. (**d**) The magnification of the selected region in (**a**). The positions of Cu and O atoms are shown in the left corner, while the cell order parameters of unit cells are retained in the rest part. (**e**) Histograms of the cell order parameters from panels a–c with the bin size *δO*_*f*_ = 0.001. Each histogram is fitted by a dashed line of the Gaussian distribution. (**f**) The mean (filled squares) and ratio between the mean and relative standard deviation (filled triangular in the inset) of the Gaussian fitting as the function of *ε*. The error bar due to the bin size is shown for each mean of the Gaussian fitting. The collective order parameters *O*_*f*_(*ε*) (open circles) in Fig. 2e are provided for comparison.

Three typical cell order parameter maps with *ε* = 0.150, 0.375, and 0.975 are displayed in Fig. 4a–c, to demonstrate the evolution of *O*_*f*_(*n*,*ε*) with the change of the reduced energy, while the full evolution is provided in Supplementary Fig. S3. At each reduced energy, the order parameter map is inhomogeneously distributed. Both positive and negative nematicities form nanoscale domains, which are depicted in red and blue colors in Fig. 4a–c. For a small reduced energy (*ε* = 0.150, in Fig. 4a), these two opposite nematic domains occupy roughly the same percentage of the image, leading to nearly zero collective nematicity. With the increase of the reduced energy, the colors of both domains are intensified so that the local nematicity is enhanced. In our selected FOV, the average strength of the positive nematicity however experiences a stronger enhancement than that of the negative nematicity. At the same time, the size of red domains grows, showing a gradual formation of the collective positive nematicity. For example, a blue domain at the top left corner of Fig. 4a is cut into two parts and a red domain emerges in Fig. 4b when the reduced energy changes from 0.150 to 0.375. On the other hand, the major domain structure is considerably preserved. With the Ising symmetry *x*_*n*_ = ± 1 assigned to a red or blue pixel for each unit cell, a correlation function, $C={\sum }_{n}|{x}_{n}-{x^{\prime} }_{n}|\,/{\sum }_{n}(|{x}_{n}|+|{x^{\prime} }_{n}|)$, is used to quantitatively describe the structural difference between the images of local order parameters at two different reduced energies. A similarity over 75% is found between Figs 4a and b. As the reduced energy further approaches the PG energy level (*ε* = 0.975, in Fig. 4c), both the size increase of red domains and the color intensification of the two domains slow down, since the cell and collective nematic orders start to be saturated. The electronic structure in overdoped Bi-2201 follows a smooth transition of symmetry breaking, and a nanoscale disorder is frozen during the establishment of the nematicity.

To gain a more quantitative description of the site-specified nematicity, we draw a histogram of *O*_*f*_(*n*,*ε*) for each reduced energy *ε* by counting *O*_*f*_(*n*,*ε*) over a selected bin size *δO*_*f*_ = 0.001. In Fig. 4e, the histograms resulted from Fig. 4a–c are plotted, each being well fitted by a Gaussian envelope, $F({O}_{f})\propto \exp [-{({O}_{f}-{O}_{f}^{G})}^{2}/2{\sigma }_{f}^{2}]$, with a mean ${O}_{f}^{G}(\varepsilon )$ and a standard deviation *σ*_*f*_. The same good accuracy of Gaussian fitting is found for all the other histograms. The center of each Gaussian distribution, ${O}_{f}^{G}(\varepsilon )$, provides another estimation of the averaged collective nematic order. As shown in Fig. 4f, the result of ${O}_{f}^{G}(\varepsilon )$ is almost the same as the collective order parameter *O*_*f*_(*ε*) defined in Eq. (2) after considering the error of the bin size. The positive collective nematic order in the selected FOV is formed by the shift of ${O}_{f}^{G}(\varepsilon )$ to the right, consistent with the gradual growth of the red domains in Fig. 4a–c. Due to its strong inhomogeneous nature, *O*_*f*_(*n*,*ε*) is always broadly distributed with a large standard deviation due to the two types of nematic domains in nanoscale. The value of *σ*_*f*_ in general increases with *ε*, as demonstrated by the gradual color intensification in Fig. 4a–c. The evolution of ${O}_{f}^{G}(\varepsilon )$ and *σ*_*f*_(*ε*) suggests that the nematic order is first randomly generated by a real-space fluctuation and then is enhanced following the increase of the reduced energy. In the development of a positive nematicity extended over a larger region in the FOV, some blue domains are sustained possibly due to a non-negligible energy cost for flipping microscopic nematic orders. The broadening of the Gaussian distribution is however less severe than the shift of its center position, with the relative ratio of ${\sigma }_{f}(\varepsilon )/{O}_{f}^{G}(\varepsilon )$ consistently reduced as *ε* increases (see the inset of Fig. 4f).

## Discussion

The STM experiment reported in this paper confirms the nematic order in overdoped Bi-2201, extending previous studies in Bi-2212^4^. The collective nematic orders with rotational symmetry breaking at O sites are observed in both momentum and real spaces. A filtering procedure allows us to extract the site-specified intra-unit-cell signal and reveal the spatial distribution of electronic nematicity. In an underdoped Bi-2212 with a strong nematicity, the dominant order can extend over a few tens of nanometers^4^. In our overdoped Bi-2201 with a weak nematicity, the two opposite orders coexist when a nonzero collective order is developed around PG. Nanoscale domains are aggregated in real space for each of two opposite nematicities, which are induced by local fluctuations. With the increase of the reduced energy, the domain size of one nematicity increases, and its order strength gradually dominates although both orders are enhanced. The collective nematic order is established by the combination of these two effects. The evolution of the nematicity in our sample can help build a multi-step picture for the order formation.

In Bi-2212, the nematic order is determined in underdoped and optimal samples, but disappears for overdoped samples without the PG state. Our observation in the overdoped Bi-2201 presents the nematicity in cuprates with a large FS, implying the nematicity more correlated with the PG state than the FS structure. On the other hand, the nematic order also coexists with other electronic orders, e.g., the charge order (smectic order) which is interpreted by a Landau-Ginzberg theory^9^. The charge order is recently identified as a *d*-form factor order with a sophisticated phase sensitive analysis^22–24^. The PG state and related charge order are believed to originate from strong-correlated doped Mott insulator^25, 26^, whereas the large FS is often related with a Fermi liquid behavior^2^. Future studies will be necessary to further explore the connections between the nematicity, charge order and FS.

## Methods

### Sample growth

The high-quality Pb-doped Bi_2_Sr_2_CuO_6+δ_ single crystals are grown by the traveling solvent floating zone method^27^. Starting materials of Bi_2_O_3_, PbO, Sr_2_CO_3_ and CuO with 99.99% purity are mixed in an agate mortar and calcined at 750 °C–810 °C in muffle furnace for 24 h. After pressed into a cylindrical rod in a hydrostatic pressure of ~ 70 MPa, the sample is sintered in a vertical molisili furnace at 840 °C for 48 h in air. The sintered rod was then pre-melted in the floating zone furnace at a traveling velocity of 25 ~ 30 mm/h to obtain a dense feed rod. By trying and optimizing the growing conditions, the dense feed rod was again melted on the seed rod just like the pre-melting process but with a slower travelling velocity of 0.5 mm/h. Single crystals of ~50 × 5 × 2 mm^3^ can be obtained by cutting from the as-grown ingot.

### STM measurement

The STM experiments are performed with an ultrahigh vacuum (UHV) and low temperature STM system. The overdoped Bi-2201 samples are cleaved in the UHV chamber at liquid nitrogen temperature (~77 K), and immediately inserted into the measurement stage. All STM results in this paper are acquired at liquid helium temperature (~4.5 K). The STM topography is typically taken with a sample bias *V* = 100 mV and a setpoint current *I* = 100 pA. The *dI*/*dV* spectra are taken with a standard lock-in technique with modulation frequency *f* = 983.4 Hz and amplitude *V*_ac_ = 3 mV. The tips are fabricated from thin Tungsten wires (0.25–0.5 mm) by electrochemical reaction recipes, and treated by e-beam sputtering and field emission cleaning on the Au (111) crystal sample.

### Data availability

The datasets generated during and/or analysed during the current study are available from the corresponding author on reasonable request.

## Electronic supplementary material

Supplementary Information
