# Supplementary material for: The study of electronic nematicity in an overdoped (Bi, Pb)2Sr2CuO6+δ superconductor using scanning tunneling spectroscopy
Source: Sci Rep. 2017 Aug 14;7:8059. doi: 10.1038/s41598-017-08376-1 (PMC5556024; doi:10.1038/s41598-017-08376-1)
Supplement: Supplementary file 1 — Supplementary Information [file 41598_2017_8376_MOESM1_ESM.pdf]

Supplementary Information for "The study of electronic nematicity in an overdoped $(Bi, Pb)_2Sr_2CuO_{6+\delta}$ superconductor using scanning tunneling spectroscopy"

Yuan Zheng[1], Ying Fei[1], Kunliang Bu[1], Wenhao Zhang[1], Ying Ding[2], Xingjiang Zhou[2,3], Jennifer E. Hoffman[4], Yi Yin[1,5,*]

1. Department of Physics, Zhejiang University, Hangzhou, 310027, China
2. Beijing National Laboratory for Condensed Matter Physics, Institute of Physics, Academy of Science, Beijing 100190, China
3. Collaborative Innovation Center of Quantum Matter, Beijing 100871, China
4. Department of Physics, Harvard University, 17 Oxford St. Cambridge, 02138, USA
5. Collaborative Innovation Center of Advanced Microstructures, Nanjing 210093, China

*Correspondence and requests for materials should be addressed to Y. Y. (email: yiyin@zju.edu.cn).

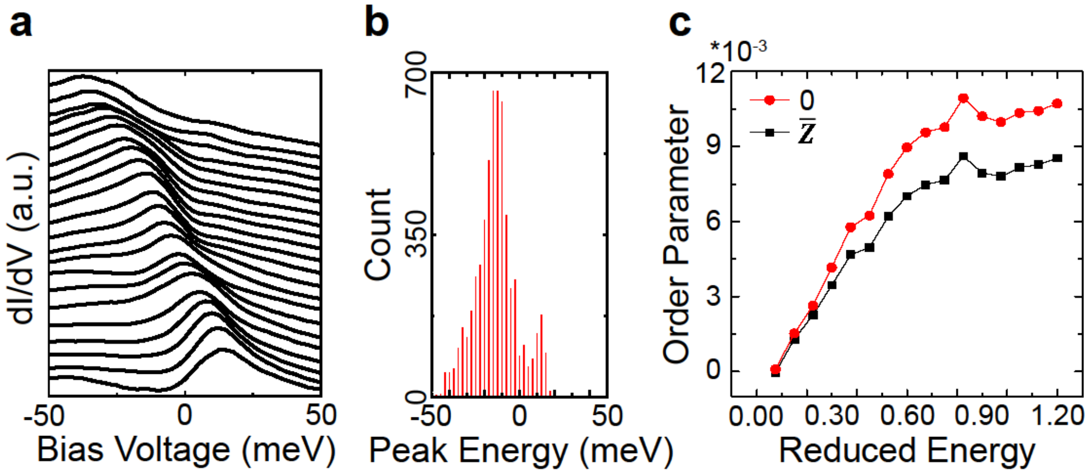

Supplementary Figure S1 | Analysis on van Hove singularity region. (**a**) The dI/dV spectra with a single peak VHS in the same FOV as in the main text. Each curve is a spatial average of all spectra sharing the same peak position of E_P binned by 2.5 meV. From top to bottom, the curves are sorted by increasing ranging from -37.5 meV to 15 meV, where a vertical offset is applied for clarity. (**b**) Histogram of the VHS peak energies. The average peak position is -10.5 meV and the standard deviation is 12.2 meV. (**c**) Reduced energy ε-dependence of the collective order parameters with the VHS region filled with zero (red curve) and $\bar{Z}$ (black curve).

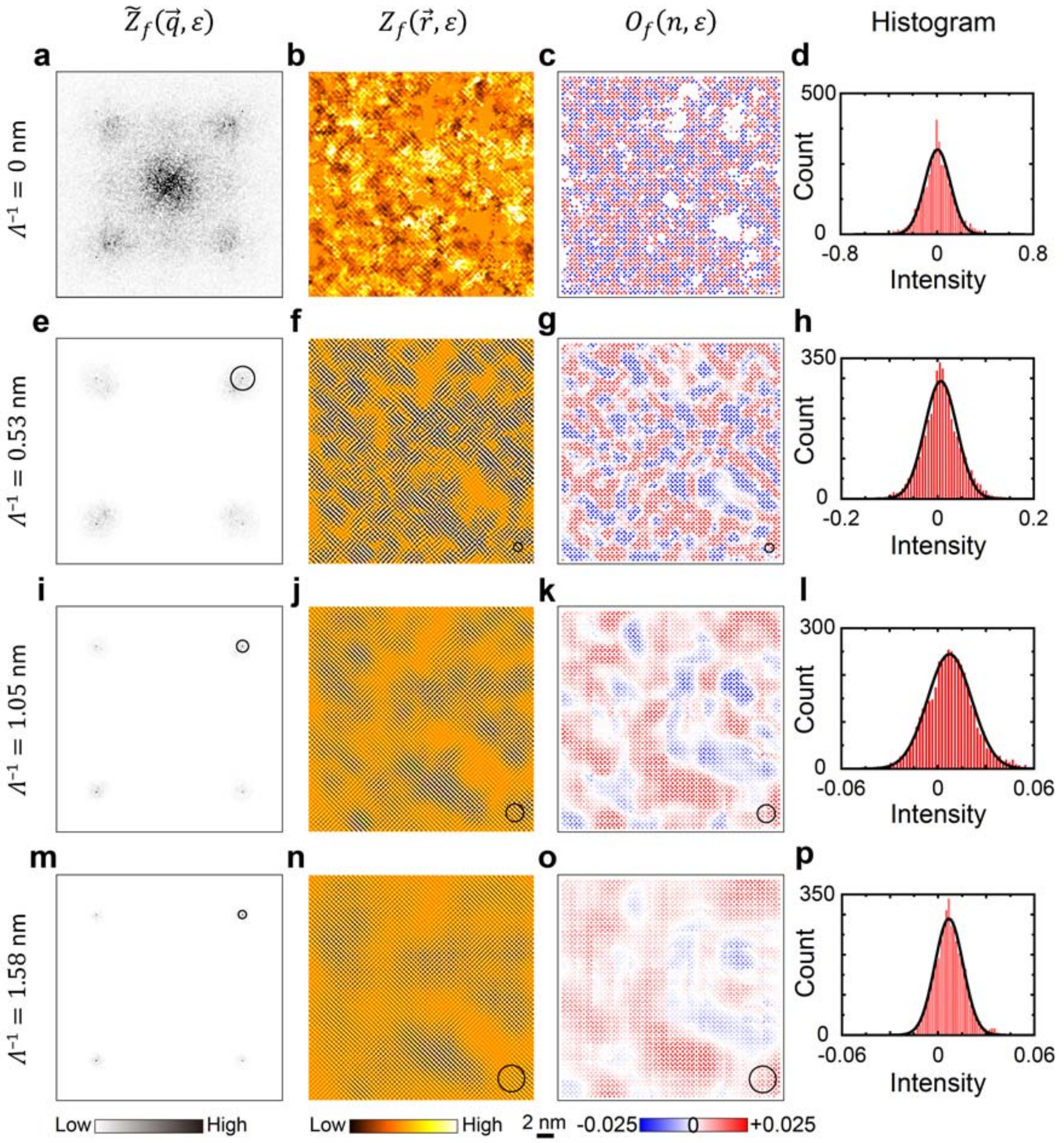

Supplementary Figure S2 | Gaussian filtering procedure for $\varepsilon = 0.975$. From left to right, figures (**a**, **e**, **i**, **m**) in the first column are the ratio maps ($\tilde{Z}(\vec{r},\varepsilon)$ and $\tilde{Z}_f(\vec{r},\varepsilon)$) in the momentum space; figures (**b**, **f**, **j**, **n**) in the second column are the ratio maps ($Z(\vec{r},\varepsilon)$ and $Z_f(\vec{r},\varepsilon)$) in the real space; figures (**c**, **g**, **k**, **o**) in the third column are the local nematic order maps ($O(n,\varepsilon)$ and $O_f(n,\varepsilon)$); figures (**d**, **f**, **j**, **n**) in the fourth column are the histogram of the corresponding cell nematic order parameters. From top to bottom, (**a**, **b**, **c**, **d**) in the first row are the unfiltered results; (**e**, **f**, **g**, **h**) in the second row are the filtered results with $\Lambda^{-1} = 0.527$ nm; (**i**, **j**, **k**, **l**) in the third row are the filtered results with $\Lambda^{-1} = 1.05$ nm; (**m**, **n**, **o**, **p**) in the fourth row are the filtered results with $\Lambda^{-1} = 1.58$ nm. The filtration size is shown by a black circle in each filtered map. For clarity of comparison, the unit cells with $O_f(n,\varepsilon) > 0.025$

and $O_f(n,\varepsilon) < 0.025$ are depicted in pure red and blue colors with the largest contrasts, respectively. The Gaussian fitting function for each histogram is represented by a solid curve.

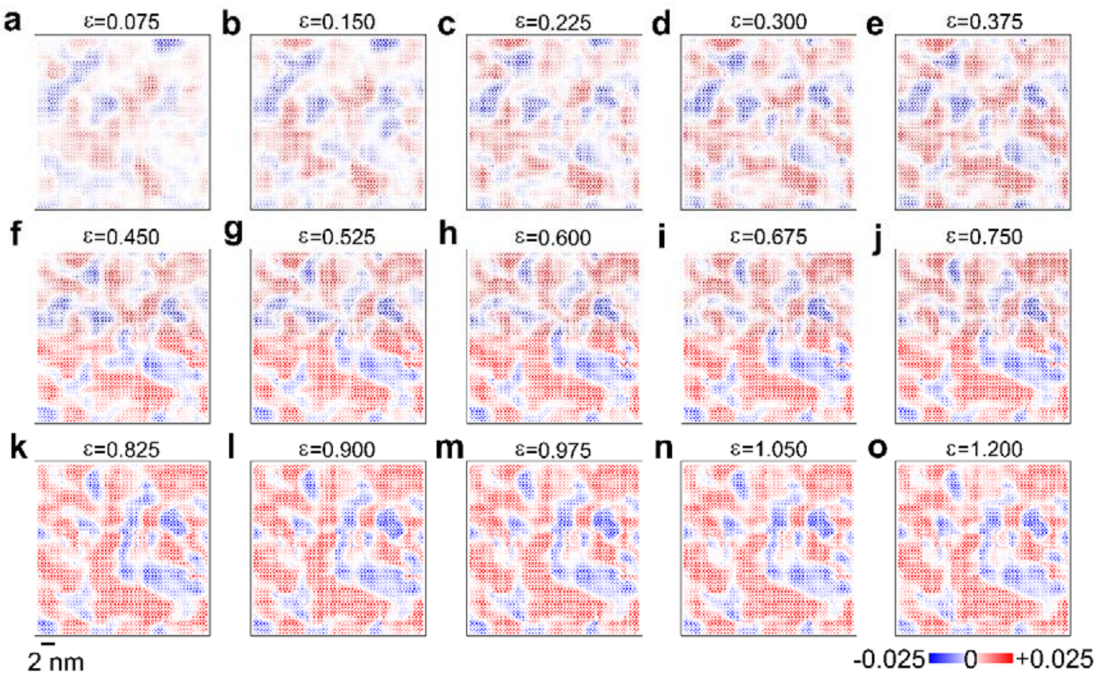

Supplementary Figure S3 | Establishment of the nematic order. (**a**)-(**o**) The evolution of the cell nematic order $O_f(n,\varepsilon)$ maps with the increase of the reduced energy from 0.075 to 1.200. All these maps are obtained under the Gaussian filtering procedure with the filtration size $\Lambda^{-1} = 1.05$ nm. For each reduced energy, the value of ε is provided for the corresponding figure. For clarity of comparison, the unit cells with $O_f(n,\varepsilon) > 0.025$ and $O_f(n,\varepsilon) < 0.025$ are depicted in pure red and blue colors with the largest contrasts, respectively.

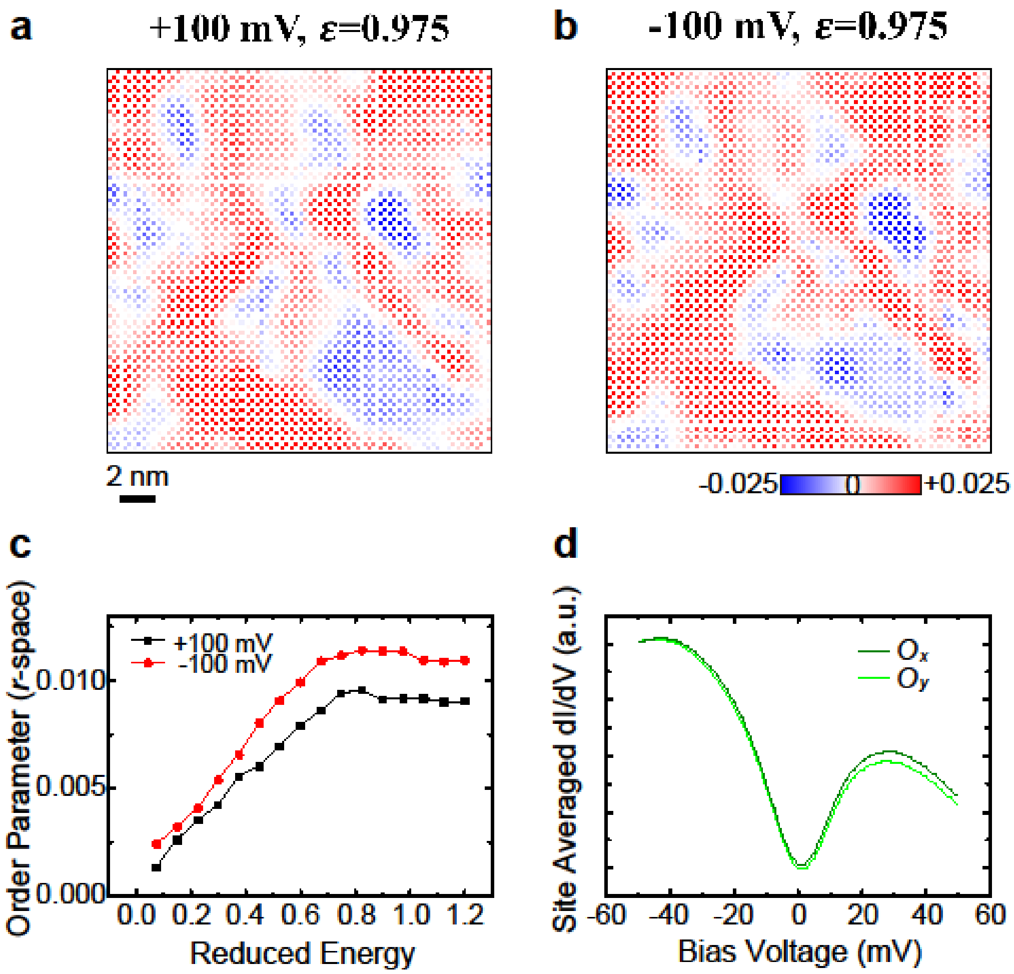

Supplementary Figure S4 | The comparison of nematic order for two datasets with opposite sample bias polarities. Maps of cell nematic order in the real space with reduced energy $\varepsilon = 0.975$, for two datasets with the sample bias voltage (**a**) $V_b = +100$ mV, and (**b**) $V_b = -100$ mV. (**c**) Reduced energy dependence of collective order parameters for both sample bias polarities. (**d**) The averaged spectra on O_x and O_y sites with the sample bias voltage $V_b = -100$ mV. With a negative sample bias, the spectral weight is normalized in the filled states (negative bias voltages), and the spectral shift mainly appears in the empty states (positive bias voltages).

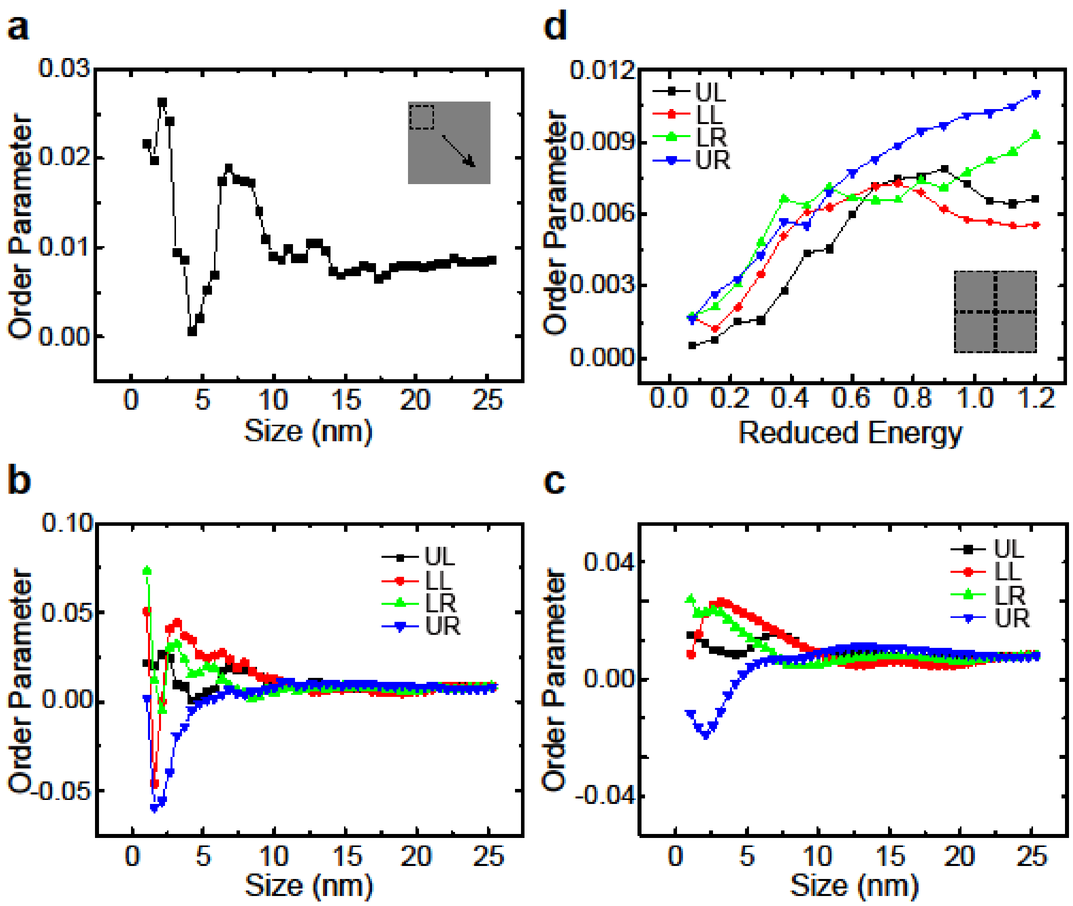

Supplementary Figure S5 | Size effect of the collective nematic order. (**a**) Size (side length) dependence of the collective order parameter of a square area. One corner of the square is fixed at the upper-left point of the same FOV as in the main text. (**b**) Size dependence of the collective nematic order extracted from the ratio map $Z(\vec{r}, \varepsilon = 0.975)$, for four different series of square areas and with corners fixed at four different points. UL, upper-left, LL, lower-left, LR, lower-right, UR, upper-right. (**c**) Size dependence of the collective nematic order similar as in panel **b**, but extracted from the filtered ratio map $Z_f(\vec{r}, \varepsilon)$. (**d**) Reduced energy dependence of the collective nematic order for four square areas at different locations, each with a size of 15×15 nm^2 (as schemed in the inset).

Supplementary Note S1 | Differential conductance spectra with a van Hove singularity

For the overdoped Bi-2201 sample studied in this paper, a pseudogap (PG) state can be determined for most of the differential conductance (dI/dV) spectra taken in the scanning field of view (FOV) (shown in Fig. 1a of the main text). Instead, a single logarithmic peak is observed around the Fermi energy level for a small portion (~10.9%) of dI/dV spectra. Such a peak is considered as the signature of a van Hove singularity (VHS) in the electronic density of state (DOS). In Fig. S1a, a series of spatially averaged VHS spectra under a bin size of $\delta E_P = 2.5$ meV are presented as the VHS peak energy E_P varies from -37.5 meV to 15 meV. As shown by the histogram in Fig. S1b, the majority of the VHS peak energies follows a distribution centered at -15 meV. However, hundreds of VHS peaks are extended to be above the Fermi energy level. In a previous study [18], the VHS spectra was found to appear as a non-cation-doped Bi-2201 sample approaches to the overdoped region. The positive PG peak is suppressed, while the original negative coherence peak is shifted to the Fermi energy level, gradually becoming a sharp VHS peak. In the whole FOV, the VHS areas are found to be surrounded by the PG areas with small gaps, and a smooth transition occurs between two characteristic spectra.

To minimize the influence of the VHS state in our analysis of the nematic order, the ratio $Z(\vec{r},\varepsilon) = g(\vec{r},\varepsilon)/\ g(\vec{r},-\varepsilon)$ for positions inside the VHS region is replaced by a mean value, $\bar{Z}(\varepsilon)$. To make sure this assumption will not affect the main conclusion of nematic order in this paper, we also assign the ratio values in the VHS region with different constants, and compare the correspondingly analyzed results. In Fig. S1c, we show the collective order as a function of the reduced energy, with the VHS region set as zero and $\bar{Z}(\varepsilon)$ respectively. There is a similar trend of how the nematicity develops. The nematicity is qualitatively not affected by the artificial treatment of VHS region in our analysis. On the other hand, although a portion of ~10.9% VHS area is discovered in the FOV analyzed in this paper, this ratio is not a general percentage of VHS region in the current sample. The detailed study of the VHS region is not explored in this paper.

Supplementary Note S2 | The Gaussian filtration procedure

A key step of analyzing the nematic order in this paper is to apply a Gaussian filtering to the ratio Z-map in the momentum space. In Fig. S2, we take a typical example around the PG energy level, $\varepsilon = 0.975$, to demonstrate this filtration procedure. Figures S2a and S2b present the original$\tilde{Z}(\vec{q},\varepsilon)$ and $Z(\vec{r},\varepsilon)$ maps, which include information of all the wavelengths in addition to the intra-unit-cell modulation. A cell nematic order, $O(n,\varepsilon) = \left[Z_y(\vec{r}_n,\varepsilon) - Z_x(\vec{r}_n,\varepsilon)\right]/\bar{Z}(\varepsilon)$, is subsequently defined for each nth unit cell in the real space. The resulting O-map is displayed in Fig. S2c, from which we observe a significant inhomogeneity of both positive and negative nematicities. The spatial average, $O(\varepsilon) = \sum_n O(n,\varepsilon)/N$, leads to a collective order, $O(\varepsilon = 0.975) = 0.008.$ The histogram of $O(n,\varepsilon)$ with the bin size $\delta O = 0.02$ in Fig. S2d is fitted by a Gaussian distribution with a mean $O^G(\varepsilon = 0.975) = 0.0052$ and a standard deviation $\sigma(\varepsilon = 0.975) = 0.107$. The atomic-site-specified nematic order is hard to be extracted from this real space distribution due to a large deviation between $O(\varepsilon)$ and $O^G(\varepsilon)$ as well as an extremely strong background noise, $\sigma(\varepsilon)/O^G(\varepsilon) \approx 20.$

Since the intra-unit-cell modulation is reflected by the Bragg peaks in the $\tilde{Z}(\vec{q},\varepsilon)$ map, we can concentrate on signals near these peaks by filtering out interference information from other spatial modulations. In practice, a smooth Gaussian cut off, $f_\Lambda(\vec{q}) = \exp(-q^2/2\Lambda^2)$, is applied to each Bragg peak, which gives rise to a filtered signal,

$$\tilde{Z}_f(\vec{q},\varepsilon) = \tilde{Z}(\vec{q},\varepsilon)\left[f_\Lambda(\vec{q}+\vec{Q}_x) + f_\Lambda(\vec{q}-\vec{Q}_x) + f_\Lambda(\vec{q}+\vec{Q}_y) + f_\Lambda(\vec{q}-\vec{Q}_y)\right].$$

Figure S2e presents a filtered $\tilde{Z}_f(\vec{q},\varepsilon)$ map under the filtration size of $\Lambda^{-1} = 0.527$ nm, which is equal to 5 pixels of the FOV (the total 256×256 pixels). Next we can take an inverse Fourier transform of $\tilde{Z}_f(\vec{q},\varepsilon)$ to obtain the filtered ratio map, $Z_f(\vec{r},\varepsilon)$, in the real space. Compared to the unfiltered map in Fig. S2b, $Z_f(\vec{r},\varepsilon)$ in Fig. S2f exhibits lattice-like patterns since a locally weighted average is considered for each spatial position. Accordingly, the filtered $O_f(n,\varepsilon)$ map in Fig. S2g is less fluctuated than the unfiltered map in Figure S2c, and inhomogeneous domains of the two opposite nematicities are observed. The parameters of the Gaussian fitting function for the histogram in Fig. S2h are $O_f^G(\varepsilon = 0.975) = 0.0067$ and $\sigma_f(\varepsilon = 0.975) = 0.034$. Although the mean value $O_f^G(\varepsilon)$ is closer to the collective nematic order, $O_f(\varepsilon = 0.975) = 0.0079$, the background noise still dominates with $\sigma_f(\varepsilon)/O_f^G(\varepsilon) \approx 5$.

Next we consider a larger filtration size, $\Lambda^{-1} = 1.05$ nm, which is equal to 10 pixels of the FOV. The maps of $\tilde{Z}_f(\vec{q},\varepsilon)$, $Z_f(\vec{r},\varepsilon)$, and $O_f(n,\varepsilon)$ following the filtering procedure are plotted in Fig. S2i, S2j, and S2k, respectively. The lattice-like patterns in $Z_f(\vec{r},\varepsilon)$ and the real-space nematic domains in $O_f(n,\varepsilon)$ are further strengthened although more information is filtered out at the same time. For the histogram in Figure S2l, the parameters of the Gaussian fitting function are $O_f^G(\varepsilon = 0.975) = 0.0074$ and $\sigma_f(\varepsilon = 0.975) = 0.014$. The mean value is nearly the same as the collective order, $O_f(\varepsilon = 0.975) = 0.0077$, and the nematic peak can be distinguished from the background noise with $\sigma_f(\varepsilon)/O_f^G(\varepsilon) \approx 2$.

The third filtration size studied is $\Lambda^{-1} = 1.58$ nm, which is equal to 15 pixels of the FOV. The maps of $\tilde{Z}_f(\vec{q},\varepsilon)$, $Z_f(\vec{r},\varepsilon)$, and $O_f(n,\varepsilon)$ following the filtering procedure are plotted in Figures S2m, S2n, and S2o, respectively. As the contrast of the $Z_f(\vec{r},\varepsilon)$ map decreases significantly, the fluctuations of local nematic domains in the $O_f(n,\varepsilon)$ map are further suppressed. For the histogram in Figure S2p, the parameters of the Gaussian fitting function are $O_f^G(\varepsilon = 0.975) = 0.0068$ and $\sigma_f(\varepsilon = 0.975) = 0.088$. Although the nematic peak is further narrowed with $\sigma_f(\varepsilon)/O_f^G(\varepsilon) \approx 1.3$, it becomes more difficult for us to extract information of atomic-site-specified nematicity, which could be essential for understanding the mechanism of nematicity.

Therefore, an appropriate filtration size Λ^{-1} is required in our analysis of the nematicity. On one hand, the filtered $Z_f(\vec{r},\varepsilon)$ map is expected to be weakly correlated with the unfiltered $Z(\vec{r},\varepsilon)$ map so that the dominant background interference signal is efficiently filtered out. On the other hand, Λ^{-1} is required to be within the average size of the electronic order, e.g., the PG state, so that the locally

weighted average is roughly taken within the area of the same Δ_{PG}. In the main text, all the filtered results are calculated using $\Lambda^{-1} = 1.05$ nm, which can satisfy both requirements and is also close to the filtration size in a previous study of Bi-2212 [3]. However, we must emphasize that the qualitative conclusion of this paper is unaffected by the choice of Λ^{-1}. The establishment of the collective nematic order $O_f(\varepsilon)$ is consistently observed as Λ^{-1} increases. In addition, a broad distribution of $O_f(n, \varepsilon)$ is sustained for a large filtration size, $\Lambda^{-1} = 1.58$ nm, which confirms the coexistence of the two opposite nematicities and their strong spatial inhomogeneity.

Supplementary Note S3 | Dataset taken with a negative sample bias voltage

As an intrinsic electronic property, the nematic order should not be dependent on the tunnel junction condition with which the spectra dataset is taken. In the same FOV as in the main text, we have changed the sample bias voltage to $V_b = -100$ mV and taken another dataset of dI/dV spectra. The similar analysis has been made to explore the nematic order. In Fig. S4a and S4b, we compare the cell nematic order maps for $V_b = +100$ mV and $V_b = -100$ mV. The similar order patterns are displayed, with very slight difference in details. The collective orders as a function of reduced energy are also compared in Fig. S4c. Qualitatively, they show the similar trend of nematicity. Furthermore, the averaged spectra on the O_x and O_y sites are compared in Fig. S4d. With the negative sample bias condition, the spectral weight is normalized for filled states (negative bias voltages) and the spectral shift mainly appears in the empty states (positive bias voltages). We consider the two detected nematic order consistent with each other, although the datasets are taken one after another and with different tunnel junction conditions.

Supplementary Note S4 | Size effect of the collective nematic order

From the cell nematic order maps we analyze and display in the main text, there is a broad real-space distribution of the site-specified nematicity, and a real space fluctuation of positive and negative regions of nematicity. The real space order fluctuation is also reproducible as shown in the supplementary note S3. With this real space fluctuation, we should reconsider how the collective order evolves as the size of the analyzed area increases. For the FOV in the main text, an area of 27×27 nm^2, we define a series of square areas with different sides, but sharing the same upper-left corner. For each square area, the collective nematic order is correspondingly calculated for the ratio maps $Z(\vec{r}, \varepsilon)$. The size dependence of the collective order is displayed in Fig. S5a. We could notice that the collective nematic order oscillates a lot for the areas with the area smaller than $\sim10\times10$ nm^2, depending on how the positive or negative regions of nematicity aggregate in the small area. But when the size increases, the collective nematic order converges to a positive value.

In Fig. S5b, we display the similar order evolution as the size increases for square areas starting from four different corners in the same FOV. The initial oscillations of the order for small square areas vary dramatically among four different series, illustrating the location dependence of evolved orders. The range of oscillation is much larger than the final converged order values, because we analyze the

data based on unfiltered ratio maps $Z(\vec{r},\varepsilon)$. The range of initial oscillation is reasonably smaller when the filtered ratio maps $Z_f(\vec{r},\varepsilon)$ are applied to obtain the size dependence of nematic order evolution, as shown in Fig. S5c.

In Fig. S5d, we roughly separate the original FOV to four different areas, each with a size of 15×15 nm^2, and display their corresponding ε-dependence of collective nematic order. They share a similar trend of nematicity, although the four areas are at different locations. With the size-effect analysis of collective nematic order as above, we consider the FOV of 27×27 nm^2 is a reasonable large enough size to define the collective nematic order. Although the quantitative value of nematic order should vary with the location and size of the FOV, we claim a qualitative discovery and mapping of nematicity in the over-doped sample in this paper.
